# The Effect on Melanoma Risk of Genes Previously Associated With Telomere Length

**DOI:** 10.1093/jnci/dju267

**Published:** 2014-09-16

**Authors:** Mark M. Iles, D. Timothy Bishop, John C. Taylor, Nicholas K. Hayward, Myriam Brossard, Anne E. Cust, Alison M. Dunning, Jeffrey E. Lee, Eric K. Moses, Lars A. Akslen, AMFS Investigators, Per A. Andresen, Marie-Françoise Avril, Esther Azizi, Giovanna Bianchi Scarrà, Kevin M. Brown, Tadeusz Dębniak, David E. Elder, Eitan Friedman, Paola Ghiorzo, Elizabeth M. Gillanders, Alisa M. Goldstein, Nelleke A. Gruis, Johan Hansson, Mark Harland, Per Helsing, Marko Hočevar, Veronica Höiom, IBD investigators, Christian Ingvar, Peter A. Kanetsky, Maria Teresa Landi, Julie Lang, G. Mark Lathrop, Jan Lubiński, Rona M. Mackie, Nicholas G. Martin, Anders Molven, Grant W. Montgomery, Srdjan Novaković, Håkan Olsson, Susana Puig, Joan Anton Puig-Butille, QMEGA and QTWIN Investigators, Graham L. Radford-Smith, Juliette Randerson-Moor, SDH Study Group, Nienke van der Stoep, Remco van Doorn, David C. Whiteman, Stuart MacGregor, Karen A. Pooley, Sarah V. Ward, Graham J. Mann, Christopher I. Amos, Paul D. P. Pharoah, Florence Demenais, Matthew H. Law, Julia A. Newton Bishop, Jennifer H. Barrett, on behalf of the GenoMEL Consortium

**Affiliations:** Affiliations of authors:Section of Epidemiology and Biostatistics, Leeds Institute of Cancer and Pathology, Leeds Cancer Research UK Centre, University of Leeds, Leeds, UK (MMI, DTB, JCT, MHa, JRM, JANB, JHB); Oncogenomics (NKH), Genetic Epidemiology (NGM), Inflammatory Bowel Diseases Laboratory (GLRS), Cancer Control Group (DCW), Statistical Genetics (SM, MHL), and Molecular Epidemiology (GWM), QIMR Berghofer Medical Research Institute, Brisbane, Australia; INSERM, UMR-946, Genetic Variation and Human Diseases Unit, Paris, France (MB, FD); Université Paris Diderot, Sorbonne Paris Cité, Institut Universitaire d’Hématologie, Paris, France (MB, FD); Cancer Epidemiology and Services Research, Sydney School of Public Health, University of Sydney, Australia (AEC); Department of Oncology, University of Cambridge, Cambridge, UK (AMD, PDPP); Department of Surgical Oncology, University of Texas MD Anderson Cancer Center, Houston, TX (JEL); Centre for Genetic Origins of Health and Disease, Faculty of Medicine, Dentistry and Health Sciences, University of Western Australia, Crawley, Australia (EKM, SVW); Centre for Cancer Biomarkers CCBIO (LAA) and Gade Laboratory for Pathology (AM), Department of Clinical Medicine, University of Bergen, Bergen, Norway; Department of Pathology, Haukeland University Hospital, Bergen, Norway (LAA); Department of Pathology, Molecular Pathology (PAA) and Department of Dermatology (PH), Oslo University Hospital, Rikshospitalet, Oslo, Norway; Assistance Publique–Hôpitaux de Paris, Hôpital Cochin, Service de Dermatologie, Université Paris Descartes, Paris, France (MFA); Department of Dermatology (EA) and Oncogenics Unit (EA, EF), Sheba Medical Center, Tel Hashomer, Sackler Faculty of Medicine, Tel Aviv, Israel (EA); Department of Internal Medicine and Medical Specialties, University of Genoa, Genoa, Italy (GBS, EFPG); Laboratory of Genetics of Rare Hereditary Cancers, San Martino-IST Research Hospital, Genoa, Italy (GBS, EFPG); Division of Cancer Epidemiology and Genetics, National Cancer Institute, National Institutes of Health, Bethesda, MD (KMB, AMG, MTL); International Hereditary Cancer Center, Pomeranian Medical University, Szczecin, Poland (TD, JL); Department of Pathology and Laboratory Medicine (DEE) and Centre for Clinical Epidemiology & Biostatistics and Department of Biostatistics & Epidemiology (PAK), Perelman School of Medicine at the University of Pennsylvania, Philadelphia, PA; Inherited Disease Research Branch, National Human Genome Research Institute, National Institutes of Health, Baltimore, MD (EMG); Department of Dermatology (NAG, RVD) and Department of Clinical Genetics, Center of Human and Clinical Genetics (NVDS), Leiden University Medical Centre, Leiden, the Netherlands; Department of Oncology-Pathology, Karolinska Institutet, Karolinska University Hospital, Solna, Stockholm, Sweden (JH, VH); Department of Surgical Oncology (MHo) and Department of Molecular Diagnostics (SN), Institute of Oncology Ljubljana, Ljubljana, Slovenia; Department of Surgery (CI) and Departments of Oncology and Cancer Epidemiology (HO), Clinical Sciences, Lund University, Lund, Sweden; Department of Public Health (RMM) and Department of Medical Genetics (JL, RMM), University of Glasgow, Glasgow, UK; McGill University and Genome Quebec Innovation Centre, Montreal, Canada (GML); Commissariat à l’Energie Atomique, Institut de Génomique, Centre National de Génotypage, Evry, France (GML); Melanoma Unit, Dermatology Department, Hospital Clinic, Institut de Investigacó Biomèdica August Pi Suñe, Universitat de Barcelona, Barcelona, Spain (SP, JAPB); CIBER de Enfermedades Raras, Instituto de Salud Carlos III, Barcelona, Spain (SP, JAPB); Department of Gastroenterology and Hepatology, Royal Brisbane & Womens Hospital, Herston, Australia (GLRS); School of Medicine, University of Queensland, Herston, Queensland, Australia (GLRS); Centre for Cancer Genetic Epidemiology, Department of Public Health and Primary Care, Strangeways Research Laboratory, Cambridge, UK (KAP, PDPP); Westmead Institute of Cancer Research, University of Sydney at Westmead Millennium Institute and Melanoma Institute Australia, Sydney, Australia (GJM); Department of Community and Family Medicine, Geisel School of Medicine, Dartmouth College, Hanover, NH (CIA).

## Abstract

Telomere length has been associated with risk of many cancers, but results are inconsistent. Seven single nucleotide polymorphisms (SNPs) previously associated with mean leukocyte telomere length were either genotyped or well-imputed in 11108 case patients and 13933 control patients from Europe, Israel, the United States and Australia, four of the seven SNPs reached a *P* value under .05 (two-sided). A genetic score that predicts telomere length, derived from these seven SNPs, is strongly associated (*P =* 8.92x10^-9^, two-sided) with melanoma risk. This demonstrates that the previously observed association between longer telomere length and increased melanoma risk is not attributable to confounding via shared environmental effects (such as ultraviolet exposure) or reverse causality. We provide the first proof that multiple germline genetic determinants of telomere length influence cancer risk.

The ends of chromosomes are protected from instability by tandem nucleotide repeats, known as telomeres. Telomeres shorten both with age and following exposures associated with cancer risk, such as smoking and ultraviolet (UV) irradiation (1,2). Thus, telomere maintenance processes are natural candidates for explaining carcinogenesis. Telomere length has been associated with risk of various age-related diseases, including cancers (3,4). However, with inconsistent results between retrospective and prospective studies (4–7) and methodological concerns (8), conclusions have been at best cautious. The recognition that any reported association might be because of either reverse causation (the cancer itself or therapeutics affecting telomere length) (9) or shared environmental factors affecting both telomere length and cancer risk has meant that the question of a causal relationship remains unresolved.

There has, however, been consistency in studies of melanoma. Longer telomeres have been associated both with increased melanoma risk in a study involving 557 cases (10), and increased nevus number (2,11), a major risk factor for melanoma (12). A prospective study of 47102 subjects (13) found no association between telomere length and overall cancer risk after adjusting for shared risk factors, although it did not account for potential differences in direction of effect by cancer site (14). However, alleles in the telomerase-coding gene *TERT* that predispose to shorter telomere length, increase the risk of most cancers but are protective for melanoma (Supplementary Materials, available online) (15). Additionally, high penetrance melanoma mutations have been reported in genes encoding components of the Shelterin complex (*POT1*), which is crucial for the maintenance and signaling function of telomeres (16): *POT1* mutations resulted in longer telomeres (17).

The existence of genetic variants influencing both telomere length and cancer susceptibility would argue against either reverse causality or shared environmental effect (the latter affecting even prospective studies), explaining the association between telomere length and cancer risk. A recent meta-analysis (18) identified seven genome-wide statistically significant loci for mean leukocyte telomere length, five (*TERC*, *TERT*, *NAF1*, *OBFC1,* and *RTEL1*) containing known telomere-related genes, and two others (*ZNF208* and *ACYP2*). Of these loci, other than *TERT*, only *TERC* and *RTEL1* have been associated with risk of any disease (18–22). The study investigated the effect of the top SNP at each of the seven loci on risk of coronary artery disease (CAD) but, despite a huge sample size (>22000 case patients and 64000 control patients), no SNP was statistically significantly associated. A score based on genotypes at these loci and effect estimates from the telomere meta-analysis showed modest association with CAD risk (*P =* .01, associating shorter telomeres with increased risk). Another study of similar design (albeit smaller and more limited coverage) (23), found genome-wide statistical significance for association between mean telomere length and *TERC*, *TERT*, *OBFC1*, a novel locus at 3p14.4, and support for *ACYP2*, *NAF1,* and *RTEL1*. Of these, only *TERT* was associated with risk of breast, ovarian, and prostate cancer, while *OBFC1* was associated with a subtype of ovarian cancer.

Given the potential role of telomere length in melanoma development, we investigated the variants identified by the telomere meta-analysis (18) in a genome-wide association study (GWAS) of melanoma. Our study consisted of 11108 case patients and 13933 control patients (Supplementary Table 1, available online) from Europe, Israel, the United States, and Australia. Written informed consent was obtained from each subject, and the investigations were performed after approval by the institutional review board for each recruiting center. As by far the biggest study of germline determinants of telomere length to date, we used the effect estimates for the seven SNPs from the telomere meta-analysis (18).

All 7 SNPs were either genotyped or well-imputed (Supplementary Materials, available online) in all melanoma GWAS samples; we tested for association between each SNP genotype and melanoma risk using SNPTEST2 (Supplementary Methods, available online) (24). Four of the seven SNPs reached nominal statistical significance, *P* values lower than .05 (rs10936599 in *TERC*, *P* = .0003; rs2736100 in *TERT*, *P* = .02; rs7675998 in *NAF1*, *P* = .03; rs9420907 in *OBFC1*, *P* = .001) (Table 1). The telomere-associated SNPs in *TERC*, *TERT*, *OBFC1,* and *RTEL1* are near (8-150kb from) SNPs strongly associated with melanoma risk (rs12696304 in *TERC*, *P* = .0001; rs455433 in *TERT*, *P* = 2.26x10^-16^; rs2995264 in *OBFC1*, *P* = 7.10x10^-6^; rs75691080 in *RTEL1*, *P* = 1.02x10^-6^) (Supplementary Figure 1, available online). Further analysis suggests the two studies may be identifying the same underlying signal in each region (Supplementary Materials, available online).

**Table 1.** Results for each telomere length-associated SNP, including effect on telomere length, melanoma risk and *P* value for melanoma association*

SNP	Chromosome	Position	Related gene	Minor allele	MAF	Telomere length beta	Melanoma beta	Melanoma *P* value^**†**^
rs10936599	3	169492101	*TERC*	T	0.252	−0.097	−0.079	.0003
rs2736100	5	1286516	*TERT*	C	0.486	0.078	0.078	.02
rs7675998	4	164007820	*NAF1*	A	0.217	−0.074	−0.063	.03
rs9420907	10	105676465	*OBFC1*	C	0.135	0.069	0.083	.001
rs8105767	19	22215441	*ZNF208*	G	0.291	0.048	0.028	.16
rs755017	20	62421622	*RTEL1*	G	0.131	0.062	0.026	.35
rs11125529	2	54475866	*ACYP2*	A	0.142	0.056	-0.004	.86

* Telomere association information and minor allele frequency taken from telomere length genome-wide association study (18). MAF = minor allele frequency; SNP = single nucleotide polymorphism.

†  Two-sided *P* values from meta-analysis of results from SNPTEST2 (24) using gene dosage and assuming an additive model.

The estimated effect of these seven SNPs on telomere length (18) and their estimated effect on melanoma risk are surprisingly well correlated (Pearson’s correlation = 0.92, *P* = .002, two-sided) (Table 1; Supplementary Figure 2, available online). For all but the least statistically significant telomere SNP (*ACYP2*), the allele associated with decreased telomere length is more frequent in control patients than melanoma case patients, consistent with a protective role for shorter telomeres in melanoma.

For each sample in our study, we constructed a genetic score predicting telomere length by calculating a weighted mean of genotype dosage across the seven telomere length–associated SNPs. The weights for each SNP were the age- and sex-adjusted effect estimates (log odds ratios) from the telomere meta-analysis (18). We then used this score in a logistic regression of melanoma risk (Supplementary Materials, available online).

We found a strong association between increased telomere score and increased risk of melanoma (*P =* 8.92×10^−9^) that was consistent across geographic regions (Figure 1). Categorizing telomere score into quartiles, we observed a linear effect on melanoma risk; those in the highest quartile are estimated to be at 1.29 times the risk of melanoma of those in the lowest quartile (Supplementary Figure 3, available online).

**Figure 1.**
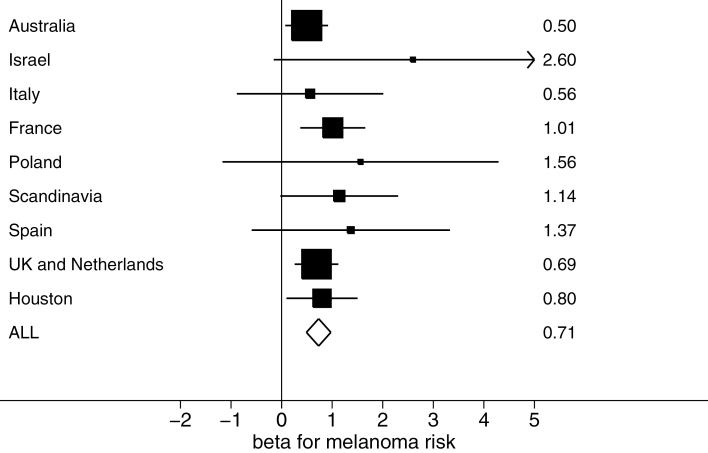
Forest plot of estimated effect size (with a 95% confidence interval indicated by **horizontal bars**) for telomere score on melanoma risk in nine geographic regions (and combined result). The relative sample size of each group is indicated by the size of the squares. Exact effect sizes (betas from SNPTEST2) are given in the right hand column.

Thus, several previously-identified telomere-associated SNPs, as well as a score based on their combined effect, are associated with melanoma risk. The fact that the telomere-associated SNP is often far less statistically significant than the strongest melanoma-associated SNP at several loci is likely in part because the telomere GWAS data are imputed from a reference panel with far fewer SNPs (Supplementary Materials, available online), so the effect of these telomere loci on melanoma risk will be underestimated here. Indeed, given the large number of genetic variants that are not able to be imputed and the possibility that several genetic variants could be responsible for the signal at a single locus, it is unlikely that the top SNP identified is a functional variant, and so the effect of the locus on both melanoma risk and telomere length is likely underestimated.

Previous studies have found at best a weak association between telomere-associated loci and disease risk. This highly statistically significant association confirms the hypothesis that the genetic factors underlying telomere length have an especially strong influence on melanoma risk and that, unusually, longer telomere length predisposes an individual to melanoma. These seven loci explained in total only 1.2% of the variation in telomere length (18), and the combined score presented here explains only 0.14% of the variation in melanoma risk (McFadden’s pseudo-r^2^). This order of magnitude is unsurprising for such a score. To put these values in context, the most statistically significant single SNP for melanoma risk in the Leeds data set is rs258322, near *MC1R* (the red hair gene); it explains 5.7% of the variation in pigmentation but only 1.29% of the variation in melanoma risk.

The biggest limitation of the present study is that it only considers the effect on melanoma risk of the seven most statistically significant loci from the telomere length GWAS, as these are the only ones for which results are publicly available. Ideally we would have included a larger number of potentially telomere-associated SNPs, rather than just those reaching genome-wide statistical significance.

Our findings do not imply that telomere length acts directly on cancer risk and could reflect pleiotropic effects of telomere-length loci (such as the ease with which telomerase is reactivated in a melanocytic nevus). However, a mechanism for melanoma has been proposed, namely that longer telomeres increase the duration of proliferation of cells in a melanocytic nevus (11). If senescence is delayed in melanocytes, this could allow further mutations to occur, increasing the chance of malignancy (10). This is the first time that a strong association between multiple telomere-associated loci and any disease risk has been established.

## Funding

The GenoMEL study (http://www.genomel.org/) was funded by the European Commission under the 6^th^ Framework Programme (contract no. LSHC-CT-2006–018702), by Cancer Research UK Programme Awards (C588/A4994 and C588/A10589), by a Cancer Research UK Project Grant (C8216/A6129), and by a grant from the US National Institutes of Health (NIH; CA83115). This research was also supported by the intramural Research Program of the NIH, National Cancer Institute (NCI), Division of Cancer Epidemiology and Genetics. Funding for the Wellcome Trust Case Control Consortium project was provided by the Wellcome Trust under award 076113.

Funding specific to particular centers is given below:

Stockholm: Swedish Cancer Society, Karolinska Institutet’s research funds.

Lund: Swedish Cancer Society, Gunnar Nilsson Foundation and European Research Council Advanced Grant (ERC-2011–294576).Genoa: Italian Ministry of Education, University and Research PRIN 2008, IMI and Mara Naum foundation. University of Genoa (PRA 2012 D31J13000000005 to PG). Intergruppo Melanoma Italiano and Mara Naum foundation to GBS

Emilia Romagna: Intramural Research Program of National Institutes of Health, National Cancer Institute, Division of Cancer Epidemiology and Genetics.

Paris: Grants from Institut National du Cancer (INCa-PL016) and Ligue Nationale Contre Le Cancer (PRE05/FD and PRE 09/FD) to FD, Programme Hospitalier de Recherche Clinique (AOM-07-195) to MFA and FD. Ligue Nationale Contre Le Cancer doctoral fellowship to MB.

Leiden: Grant provided by European Biobanking and Biomolecular Resources Research Infrastructure (BBMRI)−Netherlands hub (CO18).

Spain: The research at the Melanoma Unit in Barcelona is partially funded by Grants from Fondo de Investigaciones Sanitarias PI, 09/01393, Spain; by the CIBER de Enfermedades Raras of the Instituto de Salud Carlos III, Spain; by the AGAUR 2009 SGR 1337 of the Catalan Government, Spain.

Norway: Grants from the Comprehensive Cancer Center, Oslo University Hospital (SE0728), and the Norwegian Cancer Society (71512-PR-2006-0356).

Houston (MD Anderson): Support by the National Institutes of Health/National Cancer Institute (2P50CA093459 and P30CA023108), and by the Marit Peterson Fund for Melanoma Research.

Australian Melanoma Family Study (AMFS): AMFS is supported by the National Health and Medical Research Council of Australia (NHMRC) (project grants 566946, 107359, 211172 and program grant number 402761 to GJM and RFK); the Cancer Council New South Wales (project grant 77/00, 06/10), the Cancer Council Victoria and the Cancer Council Queensland (project grant 371); the US National Institutes of Health (via NIH RO1 grant CA-83115-01A2 to the International Melanoma Genetics Consortium - GenoMEL) and a Victorian Cancer Agency Early Career Seed Grant (ECSG07_010). AEC is supported by fellowships from the Cancer Institute NSW (10/ECF/2–06) and NHMRC (520018).

Brisbane: SM is supported by fellowships from the Australian National Health and Medical Research Council and the Australian Research Council. MHL is supported by Cancer Australia grant 1011143.

Western Australian Melanoma Health Study (WAMHS): The WAMHS gratefully acknowledges all study participants for their time and contributions, and the Western Australian DNA Bank and the Ark at the University of Western Australia for biospecimen and bioinformatics related support. The Western Australian Cancer Registry, the WAMHS study team and the WAMHS Management Committee are also gratefully acknowledged for their assistance, as well as the Scott Kirkbride Melanoma Research Centre for funding the establishment of the WAMHS resource and related salaries and PhD stipends.

Q-MEGA and QTWIN: The Q-MEGA/QTWIN studies were supported by the Melanoma Research Alliance, the NIH NCI (CA88363, CA83115, CA122838, CA87969, CA055075, CA100264, CA133996, and CA49449), the National Health and Medical Research Council of Australia (NHMRC) (200071, 241944, 339462, 380385, 389927, 389875, 389891, 389892, 389938, 443036, 442915, 442981, 496610, 496675, 496739, 552485, 552498), the Cancer Councils New South Wales, Victoria and Queensland, the Cancer Institute New South Wales, the Cooperative Research Centre for Discovery of Genes for Common Human Diseases (CRC), Cerylid Biosciences (Melbourne), the Australian Cancer Research Foundation, the Wellcome Trust (WT084766/Z/08/Z), and donations from Neville and Shirley Hawkins.

QIMR Endometriosis study: The QIMR Study was supported by grants from the National Health and Medical Research Council (NHMRC) of Australia (496610), the Cooperative Research Centre for Discovery of Genes for Common Human Diseases (CRC) and Cerylid Biosciences (Melbourne). Endometriosis sample genotyping was funded by grants from the NHMRC (496610) and Wellcome Trust (WT084766/Z/08/Z). GWM was supported by an NHMRC Fellowship (339446, 619667).

Study of Digestive Health (SDH): The SDH was supported by the National Cancer Institute (5 RO1 CA 001833-02).

Inflammatory Bowel Disease study (IBD): This research was supported by the United States National Cancer Institute (grant number CA 001833-03). DCW is a Senior Research Fellow of the National Health and Medical Research Council of Australia. NP was supported by a PhD scholarship from the National Health and Medical Research Council of Australia.

## Notes

The study sponsors had no role in the design of the study, the collection, analysis, or interpretation of the data, the writing of the manuscript, nor the decision to submit the manuscript for publication.

This study makes use of data generated by the Wellcome Trust Case Control Consortium. A full list of the investigators who contributed to the generation of the data is available on their website (http://www.wtccc.org.uk/).

The authors thank the Epidemiological Study on the Genetics and Environment of Asthma (EGEA) Cooperative Group for giving access to data of the EGEA study. We acknowledge that the biological specimens of the French Familial Melanoma Study Group were obtained from the Institut Gustave Roussy and Fondation Jean Dausset–CEPH Biobanks.
